# Clinical report and predictors of sequelae of 319 cases of pediatric bacterial osteomyelitis

**DOI:** 10.1038/s41598-022-19208-2

**Published:** 2022-09-01

**Authors:** Andrzej Krzysztofiak, Marco Roversi, Antonio Musolino, Marco Cirillo, Renato Maria Toniolo, Osvaldo Mazza, Livia Gargiullo, Laura Lancella, Paolo Rossi, Alberto Villani, Collaborative Osteomyelitis Study Group, Domenico Barbuti, Stefania Bernardi, Paola Bernaschi, Francesco Biagiarelli, Elena Boccuzzi, Elena Bozzola, Francesca Ippolita Calò Carducci, Sara Chiurchiù, Marco Crostelli, Laura Cursi, Maia De Luca, Martina Di Giuseppe, Fabrizio De Benedetti, Daniele Deriu, Marco Giordano, Annalisa Grandin, Antonella Insalaco, Elena Inzaghi, Andrzej Krzysztofiak, Alessandra Marchesi, Maria Rosaria Marchili, Gianluca Mirra, Antonio Musolino, Gian Luigi Natali, Valeria Pansini, Massimo Fabio Pezzoli, Lorenza Romani, Lelia Rotondi Aufiero, Marco Roversi, Isabella Tarissi De Iacobis, Anna Chiara Vittucci

**Affiliations:** 1 Pediatric and Infectious Disease Unit, Academic Department of Pediatrics, Bambino Gesù Children’s Hospital, IRCCS, Rome, Italy; 2 Academic Department of Pediatrics, Bambino Gesù Children’s Hospital, IRCCS, Rome, Italy; 3 University of Rome Tor Vergata, Rome, Italy; 4 Department of Imaging, Bambino Gesù Children’s Hospital, IRCCS, Rome, Italy; 5 Traumatology Unit, Surgery Department, Bambino Gesù Children’s Hospital, IRCCS, Rome, Italy; 6 Spine Surgery Unit, Department of Surgery and Transplantations, Bambino Gesù Children’s Hospital, IRCSS, Rome, Italy; 7 Department of Emergency, Acceptance and General Pediatrics, Bambino Gesù Children’s Hospital, IRCCS, Rome, Italy; 8 Unit of Microbiology and Diagnostic Immunology, Department of Diagnostic and Laboratory Medicine, Bambino Gesù Children’s Hospital, IRCCS, Rome, Italy; 9 Division of Rheumathology, Bambino Gesù Children’s Hospital, IRCCS, Rome, Italy; 10 Pediatric Pulmonology & Respiratory Intermediate Care Unit, Sleep and Long-Term Ventilation Unit, Academic Department of Pediatrics, Bambino Gesù Children’s Hospital, IRCCS, Rome, Italy

**Keywords:** Medical research, Health occupations

## Abstract

Pediatric osteomyelitis is an insidious disease that can lead to permanent sequelae, the management of which still relies on lengthy intravenous antibiotic therapy. The purpose of this study is to report and describe the clinical course and outcome of pediatric bacterial osteomyelitis in our experience. We reported the clinical, diagnostic, and treatment characteristics of all cases of osteomyelitis in children younger than 18 years of age who were hospitalized between January 2010 and December 2021 at the Bambino Gesù Children’s Hospital in Rome, Italy, we compared patients with and without complications at follow-up, to identify any predictive factor for sequelae. The study sample included 319 cases of pediatric bacterial osteomyelitis. The median age was 7.77 years. Males (60.8%) were more affected than females. The most affected bones were the femur, tibia, and spine. Etiology was identified in 40.1% of cases, with *S.aureus* as the most common causative agent. Sequelae were reported in 43 cases (13.5%). The main predictors of sequelae were sepsis on admission and hypergammaglobulinemia. Our results show that a severe presentation with sepsis and hypergammaglobulinemia on admission may be associated with a higher frequency of late sequelae. Early recognition and aggressive treatment of this subgroup of patients may lead to a reduction in complications.

## Introduction

The reported incidence of bacterial osteomyelitis in the pediatric population in developed countries is about 13 cases per 100,000 children, with higher rates in males younger than 5 years of age^1,2^. Long bones, particularly the humerus, femur, and tibia, are the most frequently affected skeletal bones, followed by the pelvic girdle and spine^3^. The clinical presentation and course of pediatric bacterial osteomyelitis depends largely on the site and characteristics of the affected bone; in addition, it is also important to consider the age group of the patient, because of the different disorders and varying bacterial etiology according to age. In this regard, pediatric spondylodiscitis and neonatal osteomyelitis deserve separate discussion: afebrile back pain may be the only manifestation of pediatric spondylodiscitis^4^, while in infants an insidious onset with inconsolable crying may anticipate swelling or reduced mobility of affected limbs, despite an underlying multifocal infection that is often severe^5^. Because of abscess formation and sequestration of infected material within the medullary of long bones, osteomyelitis is unlikely to resolve without appropriate therapy. Therefore, subacute and chronic forms may be the result of delayed antibiotic treatment or non-response to antibiotics. In the latter case, comorbidities such as immunodeficiencies^6^ and sickle cell disease^7^, which favor the rooting of unusual causative pathogens in bone, including fungi and protozoa, must be excluded. Laboratory workup of pediatric bacterial osteomyelitis should always include serial assessments of CBC and inflammatory biomarkers (particularly CRP and ESR), as well as microbial cultures, despite the very low diagnostic yield of the latter, never exceeding 50 percent^8^. *S.aureus* is isolated from about 78% of all positive cultures^9^. Other causative agents should be suspected in younger patients, namely *S.agalactiae* or gram-negative rods in infants younger than 2 months and streptococci or *K.kingae* in children younger than 5 years^10^. Imaging of the infected site is also helpful in guiding diagnosis and therapy. Radiographs can identify areas of bone resorption 2 to 3 weeks after the onset of symptoms, or a fracture in post-traumatic cases, or even cortical irregularities that are more common in malignancies that mimic osteomyelitis, such as Ewing's sarcoma^3^. In contrast, MRI can detect signs of bone edema 24 to 48 h after the onset of symptoms, with high sensitivity and specificity for pediatric bacterial osteomyelitis^11^. When joint effusion, soft tissue abscess, and subperiosteal collection are suspected, ultrasound of the affected site should always be performed^12^. Scintigraphy and CT scan are reserved for doubtful cases of multifocal involvement, noninfectious etiology, and surgical preparation. The main treatment of pediatric bacterial osteomyelitis is intravenous antibiotics, mainly beta-lactams such as cephalosporins and penicillins to cover the predominant etiology of gram-positive strains. However, the duration of therapy is still debated internationally. Historically, children with bacterial osteomyelitis have been given intravenous antibiotics for 2–4 weeks, followed by 3–4 weeks of oral antibiotic therapy. Recently, an early switch to oral antibiotics has been proposed, based on clinical and laboratory indices of remission^13^. Surgical intervention is necessary in complicated osteomyelitis (about 10% of cases) when medical treatment fails^3^. Severe adverse sequelae of pediatric bacterial osteomyelitis range from 7.9 to 29% across cohorts and countries, with a higher prevalence in younger children with a severe clinical presentation^14,15^.

All of the above are analyzed and discussed in this retrospective study, in which demographic and clinical data on 319 cases of pediatric bacterial osteomyelitis are reported, with the aim of identifying subgroups of patients who may benefit from selected management strategies, based on their sequelae and predictors of sequelae.

## Methods

All patients younger than 18 years of age admitted to Bambino Gesù Children’s Hospital (Rome, Italy) between January 2010 and December 2021 with a diagnosis of pediatric bacterial osteomyelitis were included in the study.

### Definition of osteomyelitis

Osteomyelitis is an inflammatory process accompanied by bone destruction, most frequently caused by bacteria that spread to the bone marrow and tissues near the bone^16^. Therefore, pediatric bacterial osteomyelitis was diagnosed when clinical signs or symptoms of inflammation localized to a skeletal segment and at least one radiologic sign of bone destruction and/or resorption were present in children younger than 18 years, in the absence of another plausible diagnosis. Osteomyelitis can be further classified into acute or chronic and uncomplicated or complicated^9^. Acute osteomyelitis is diagnosed within 4 weeks of onset, whereas chronic infection is a more prolonged, often indolent disease process with sequestration and/or recurrence at the same site weeks to years after treatment of the initial infection at that site. Any osteomyelitis that involves two or more bones, spreads to the physis and adjacent joint or soft tissues, and/or requires further diagnostic or therapeutic interventions may be classified as complicated^9^.

### Patients’ data

The following variables were reported: age at onset; age groups (< 1 year; 1 to 4.99 years; 5 to 10.99 years; 11 to 17. 99 years); gender; ethnicity; signs and symptoms at onset; time from onset to admission; diagnosis at admission; comorbidities; history of skin lesion, primary bone injury or trauma; blood parameters at onset, including absolute white blood cell count, absolute count and percentage of neutrophils (N), erythrocyte sedimentation rate (ESR, mm/h), C-reactive protein (CRP, mg/dl), immunoglobulin IgA, IgG, IgM levels; radiologic imaging, including radiographs, ultrasound, computed tomography (CT), magnetic resonance imaging (MRI), scintigraphy; presence of perilesional cellulitis, myositis, soft tissue abscess, or bone sequestration; duration of intravenous (IV), oral (OS), and total antibiotic therapy; history of surgery; duration of hospitalization; duration of follow-up; and presence or absence of recurrence, sequelae, or chronic osteomyelitis. A sequela was defined by the development of a clinically relevant impairment—mainly reduced range of motion of the adjacent joint, lameness, or permanent deformity—not present before the osteomyelitis episode. We also reported: affected bones; etiology (when available); classes of antibiotics used. In addition, we compared the clinical and demographic characteristics of patients who did or did not subsequently develop sequelae. Finally, we sought to identify significant clinical and/or demographic predictors of sequelae.

### Statistical analysis

IBM SPSS version 23.0 software was used for statistical analysis. Continuous variables with normal distribution were expressed as means and standard deviations. Continuous variables with non-normal distribution were expressed as medians, first and third quintiles and ranges and analyzed with the Mann–Whitney U-test. Categorical variables were expressed as proportions and percentages and analyzed with the chi-square test or Fisher's exact test (when appropriate). Logistic regression analysis was performed to identify significant predictors of the sequelae, treated as dichotomous variables. ROC curve analysis was performed on the significant variables in the multivariate analysis to identify clinically valid cutoffs. A *p* value < 0.05 was considered statistically significant.

### Ethical approval and informed consent

All study procedures were performed in accordance with current guidelines and regulations. Parents or legal guardians of study participants signed an informed consent form upon admission to the hospital for anonymous use of their data for research purposes. The study was approved by the Ethics and Scientific Committee of the Bambino Gesù Children’s Hospital.

## Results

The demographic and clinical characteristics of the study sample are outlined in Table 1.

**Table 1:** Clinical and demographic characteristics of the study sample.

Total	319
Age (years)–median; Q1-Q3 (range) < 0.99 year–no. (%) 1–4.99 years–no. (%) 5–10.99 years–no. (%) 11–17.99 years–no. (%)	7.77; 1.86–11.72 (0.01–17.77) 39 (12.2) 93 (29.2) 102 (32.0) 85 (26.6)
Males–no. (%) Male to female ratio	194 (60.8) 1:0.6
**Onset signs and symptoms** Fever–no. (%) Pain/loss of function–no. (%) Redness–no. (%) Heat–no. (%) Swelling–no. (%) Sepsis–no. (%)	181 (56.7) 303 (95.0) 124 (38.9) 146 (45.8) 173 (54.2) 52 (16.3)
Onset to referral (days)–median; Q1–Q3 (range) < 1 week–no. (%) 1–2 weeks–no. (%) > 2 weeks–no. (%)	7; 3–27 (0–1280) 198 (62.1) 70 (21.9) 51 (16.0)
**Diagnosis on admission** Osteomyelitis–no. (%) Arthritis–no. (%) Arthralgia–no. (%) Limp–no. (%) Spondylodiscitis–no. (%) Back pain–no. (%) Other–no. (%)	93 (29.2) 67 (21.0) 28 (8.8) 20 (6.3) 11 (3.4) 9 (2.8) 91 (28.5)
Comorbidities–no. (%) Fracture Infections Hematologic disease Fracture or other disease requiring surgery	69 (21.8) 15 (4.7) 14 (4.4) 14 (4.4) 11 (3.4)
Skin lesion–no. (%)	36 (11.3)
Primary bone lesion^1^–no. (%)	6 (1.9)
History of trauma–no. (%)	82 (25.7)
**Blood parameters** White blood cells–median; Q1–Q3 (range) Neutrophils (%)– median; Q1–Q3 (range) Neutrophils (count)–median; Q1–Q3 (range) ESR (mm/h)–median; Q1–Q3 (range) CRP (mg/dl)–median; Q1–Q3 (range) IgA (g/l)–median; Q1–Q3 (range) IgG (g/l)–mean; SD (range) IgM (g/l)–median; Q1–Q3 (range)	9970; 7320–13,770 (2000–45,860) 0.56; 0.41–0.72 (0.15–0.92) 5190; 3300–8016 (636–37,422) 35; 19–53 (0–124) 3.0; 0.6–8.3 (0.0–87.0) 1.15; 0.70–11.4 (0.04–9.12) 9.51; 4.37 (0.38–34.64) 1.09 (0.04–7.00)
**Radiological imaging** X-ray–no. (%) MRI–no. (%) Ultrasound–no. (%) Arthritis–no. (%) Scintigraphy–no. (%) Biopsy–no. (%) CT-scan–no. (%)	307 (96.2) 292 (91.5) 182 (57.1) 151 (47.3) 123 (38.6) 63 (19.7) 54 (16.9)

^1^In more detail: avascular necrosis of the femoral head (*n* = 1); diffuse osteopenia (*n* = 1); aneurysmal bone cyst (*n* = 1); osteochondroma (*n* = 1); Blount disease (*n* = 1), Kohler disease (*n* = 1).

### Demographics

In total, 319 cases of pediatric bacterial osteomyelitis were reported. The median age was 7.77 years (IQR 1.86 to 11.72). Apart from infants (12.2%), patients were evenly distributed in the selected age groups, with the cohort aged 5 to 10.99 years being the most represented (32%). Males (60.8%) were more affected than females, with a female-to-male ratio of 1:1.7.

### Clinical presentation

The most frequent symptom at onset was pain with loss of function of the infected limb (95.0%). More than half of the subjects had fever on admission (56.7%) with palpable swelling (54.2%) and warmth (45.8%) at the affected site. The median time from onset to admission was 7 days, with most patients admitted within 2 weeks of onset (84.0%). The most frequent diagnoses at admission were osteomyelitis (29.2%) or arthritis (21.0%). Of the study sample, 21.8% had some comorbidity, 25.7% had a history of trauma prior to the onset of osteomyelitis, 4.7% had a fracture, and 3.4% had undergone bone surgery for any reason.

### Laboratory workup

**Figure 1:**
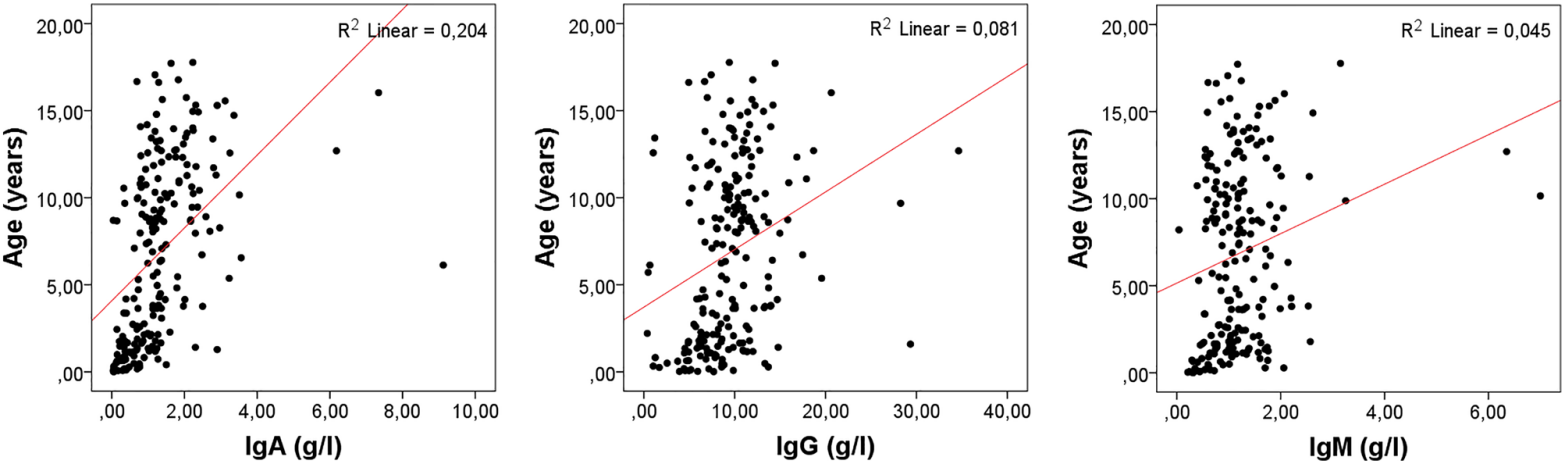
Plot of IgA, IgG, and IgM levels (g/l) against age (years) ^a^Acromion n = 1; clavicle n = 5; scapula n = 1 ^b^Fingers *n* = 4; metacarpal bones n = 2 ^c^Cuboid *n* = 1; cuneiform *n* = 2; navicular *n* = 1. ^d^Metatarsal bones n = 6; toes *n* = 17.

On admission, laboratory analysis showed median white blood cell and neutrophil counts in the normal range (9,970 and 5,190 cells/mm3, respectively). Median levels of erythrocyte sedimentation rate (ESR) and C-reactive protein (CRP) were slightly elevated (35 mm/h and 3.0 mg/dl, respectively). The median levels of IgA and IgM were 1.15 and 1.09 g/l, respectively. Median IgG levels were 9.51 g/l. Because immunoglobulin levels are known to vary widely in pediatrics, we plotted the level of IgA, IgG and IgM against age, but found a non-significant correlation, as shown in Fig. 1.

### Radiological imaging

The vast majority of patients initially underwent standard radiography (96.2%), with antero-posterior and lateral projections, followed by contrast-enhanced MRI (91.5%). Ultrasound of the hip joint was frequently performed (57.1%), identifying signs suggestive of arthritis in 47.3% of cases. CT-guided biopsy of the lesion was performed (19.7%) when, in the absence of a microbiological isolate, intravenous broad-spectrum antibiotic therapy did not improve laboratory tests within 2 weeks. The most common local complication was a soft tissue abscess adjacent to the infected focus (43.9%), followed by perilesional cellulitis (37%).

### Sites of infection and etiology

**Figure 2:**
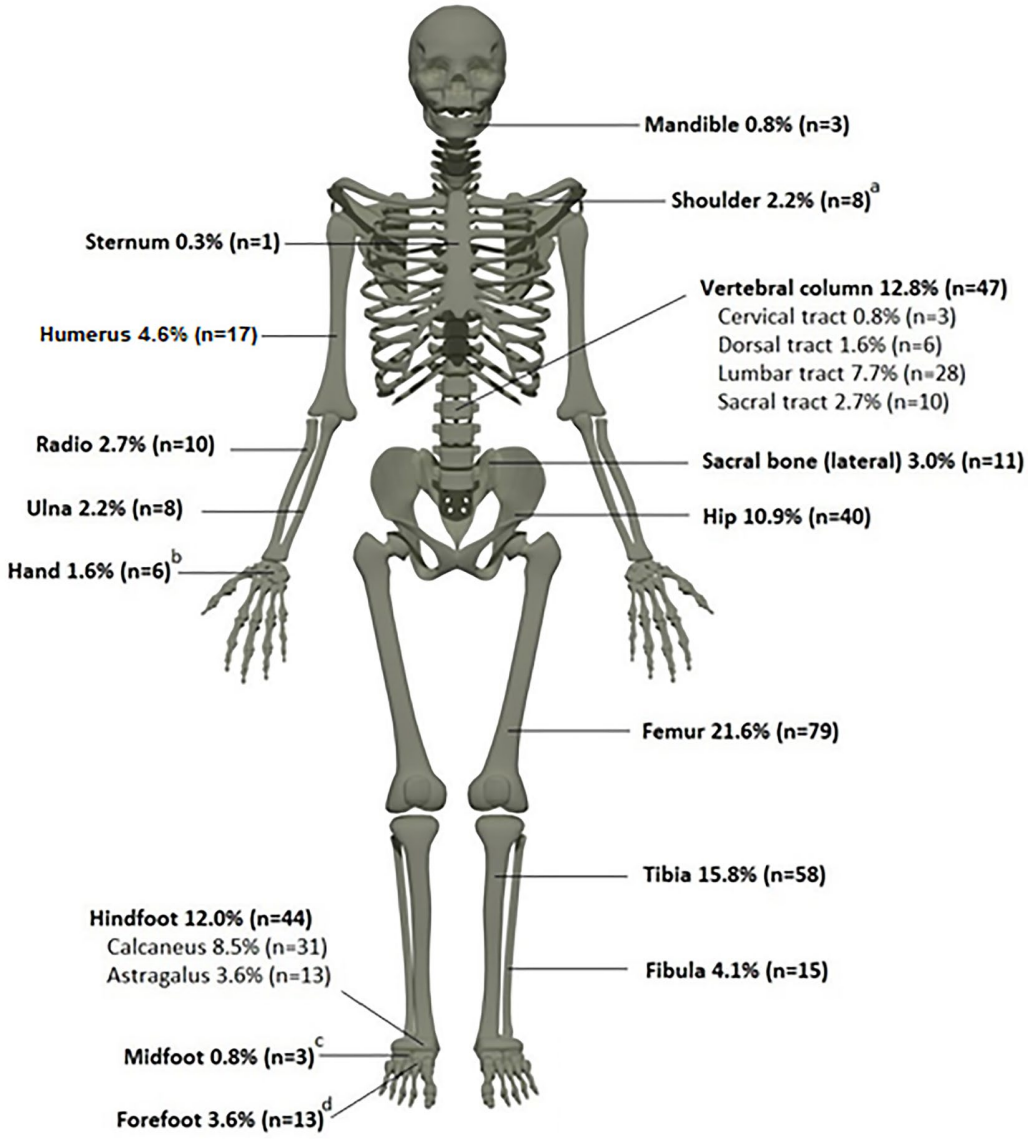
Sites of bone infection Modified from: File:201,805 human skeleton.png. DataBase Center for Life Science (DBCLS), CC BY 4.0 < https://creativecommons.org/licenses/by/4.0 > , via Wikimedia Commons Available at: https://upload.wikimedia.org/wikipedia/commons/5/5c/201805_human_skeleton.png.

The sites of infection are outlined in Fig. 2.

The most common sites of infection were the femur (21.6%), tibia (15.8%) and spine (12.8%). In the spine, mainly the lumbosacral tracts were involved (10.4%). Other commonly affected bones were the hind foot (12.0%) and the hip bone (10.9%). Of note, coinfection of the calcaneus and talus bones, as well as the sacrum (lateral sides) and hip bones, was frequently reported.

**Figure 3:**
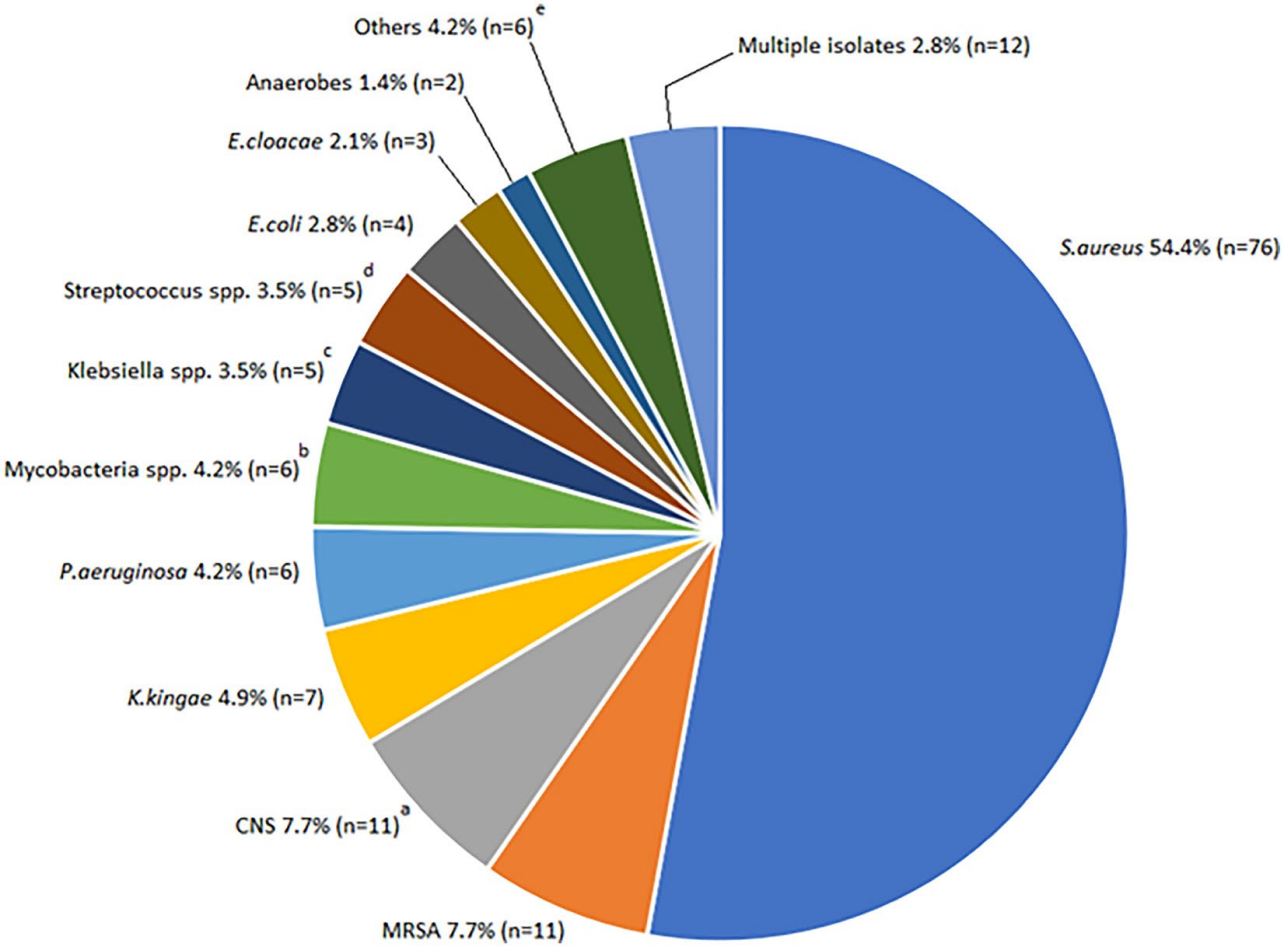
Infectious etiology of osteomyelitis in the study sample ^a^*S.epidermidis* n = 2; *S.hominis n* = 4; *S.intermedius* n = 4; *S.warneri n* = 2 ^b^*M.tuberculosis* n = 5; *M.xenopi n* = 1 ^c^*K.pneumoniae n* = 4 (MDR *n* = 1); *K.oxytoca n* = 1 ^d^*S.mitis n* = 1; *S.oralis n* = 1; *S.pneumoniae n* = 1; *S.pyogenes n* = 2. ^e^*A.baumanii n* = 1; *A.odontolyticyus n* = 1; *C.albicans n* = 1; *E.faecalis n* = 1; *P.mirabilis* n = 1; Salmonella spp. *n* = 1.

The available microbiological isolates are shown in Fig. 3.

Samples of blood or other biological material (saliva, urine, or pus from the infected site) were sent for bacterial culture in all patients, but were positive in 128 cases (40.1%). The most commonly detected pathogens were methicillin-sensitive *S.aureus* or MSSA (54.0%), methicillin-resistant *S.aureus* or MRSA (7.7%), coagulase-negative staphylococci or CoNS (7.7%) and *K.kingae* (4.9%). Of all isolated *S.aureus*, 3 (3.4%) were *Panton-Valentin Leukocidin* (PVL) producing strains. Among the remaining bacteria, we found a multi-drug resistant *K.pneumoniae*, which was sensitive only to aminoglycosides. More than one bacterial species was isolated in 12 cases (3.8%).

### Treatment, follow-up, and sequelae

Data about treatment, follow-up and sequelae are outlined in Table 2.

**Table 2:** Treatment, follow-up, complications and sequelae.

IV antibiotics Cephalosporins–no. (%) Linezolid–no. (%) Glycopeptides–no. (%) Aminoglycosides–no. (%) Meropenem–no. (%) Amoxicillin-clavulanate–no. (%) Rifampicin–no. (%) Quinolones–no. (%) Fosfomycin–no. (%)	219 (68.7) 112 (35.1) 103 (32.3) 77 (24.1) 35 (11.0) 33 (10.3) 25 (7.8) 20 (6.3) 13 (4.1)
OS antibiotics Quinolones–no. (%) Linezolid–no. (%) Amoxicillin-clavulanate–no. (%) Cephalosporins–no. (%) Rifampicin–no. (%) Clindamycin–no. (%) IREP–no. (%) Glycopeptides–no. (%)	123 (38.6) 79 (24.8) 75 (23.5) 43 (13.5) 17 (5.3) 7 (2.2) 7 (2.2) 6 (1.9)
IV therapy (days)–median (range) < 14 days–no. (%) > 14 days–no. (%) OS therapy (days)–median (range) < 14 days–no. (%) > 14 days–no. (%) Total antibiotic therapy (days)–median (range) < 6 weeks–no. (%) > 6 weeks–no. (%)	21 (14–29) 58 (18.2) 261 (81.8) 16 (12–24.5) 92 (28.8) 227 (71.2) 42 (31–49) 305 (95.6) 12 (3.8)
Local complications Soft-tissue abscess–no. (%) Perilesional cellulitis–no. (%) Myositis–no. (%) Bone sequestration–no. (%)	139 (43.6) 118 (37) 67 (21.0) 40 (12.5)
Surgical intervention Curettage–no. (%) Arthrocentesis–no. (%) Osteosynthesis–no. (%)	40 (12.5) 11 (3.4) 12 (3.8)
Hospitalization (days)–median (Q1-Q3)	22.5 (16–31)
Follow-up–median; Q1–Q3 (range)	8; 4–14 (0–60)
Relapse–no. (%)	17 (5.3)
Sequelae–no. (%)	43 (13.5)
Chronic osteomyelitis–no. (%)	45 (14.1)

All patients were started on admission with broad-spectrum intravenous antibiotic therapy, which was subsequently modified according to the patient's clinical response and isolates. The most frequently administered intravenous antibiotics were cephalosporins (68.7%) and linezolid (35.1%), followed by glycopeptides (32.3%) and aminoglycosides (24.1%). The most frequently administered oral antibiotics were quinolones (38.6%), linezolid (24.8%) and amoxicillin-clavulanate (23.5%). Most patients were treated with intravenous antibiotics during hospitalization (81.8%) and with oral antibiotics in the outpatient setting (71.2%) for more than 14 days. The total duration of antibiotic therapy did not exceed 6 weeks in most cases (95.6%). Surgery was required in a minority of cases, with curettage needed in 40 cases (12.5%). The median length of hospitalization was 22.5 days. The median follow-up time was 8 months. All patients not lost to follow-up were studied with contrast-enhanced MRI at 1, 3, 6 and 12 months. Sequelae were reported in 43 cases (13.5%). A relapse requiring rehospitalization was observed in 17 cases (5.3%). In 45 patients (14.1%) chronic osteomyelitis was detected on admission or at follow-up visits. All patients survived.

### Predictors of sequelae

The comparison of clinical and demographic characteristics of patients who subsequently developed sequelae or not is shown in Table 3.

**Table 3:** Comparison of patients with and without sequelae.

	No sequelae	Sequelae	*p-value*
Total*	258	43	−
Age (years)–median (Q1-Q3)	7.38 (1.77–11.13)	8.33 (3.64–13.69)	0.113
< 0.99 year–no. (%)	33 (12.8)	4 (9.3)	0.519
1–4.99 years–no. (%)	77 (29.8)	12 (27.9)	0.797
5–10.99 years–no. (%)	83 (32.2)	10 (23.3)	0.241
11–17.99 years–no. (%)	65 (25.2)	17 (39.5)	0.051
Males–no. (%)	157 (60.9)	27 (62.8)	0.809
**Onset signs and symptoms**			
Fever–no. (%)	140 (54.3)	27 (62.8)	0.298
Sepsis–no. (%)	36 (14.0)	15 (34.9)	**0.001**
Onset to referral (days)–median (Q1–Q3)	7 (3–25)	11 (3–60)	0.075
Comorbidities–no. (%)	56 (21.7)	10 (23.3)	0.820
Skin lesion–no. (%)	28 (10.9)	5 (11.6)	0.797
Primary bone lesion–no. (%)	6 (2.3)	0 (0)	0.312
History of trauma–no. (%)	67 (25.9)	11 (25.6)	0.987
**Blood parameters–median (Q1–Q3)**			
White blood cells	9860 (7320–13,707)	9470 (6640–14,350)	0.772
Neutrophils (%)	0.55 (0.41–0.71)	0.62 (0.42–0.73)	0.353
Neutrophils (count)	5129 (3316–7709)	5123 (2956–10,375)	0.885
ESR (mm/h)	33 (18–50)	50 (18–64)	0.139
CRP (mg/dl)	2.9 (0.6–7.9)	3.5 (0.7–11.3)	0.534
IgA (g/l)	1.14 (0.68–1.62)	1.25 (0.77–2.23)	0.347
IgG (g/l)	8.94 (6.70–11.05)	10.6 (8.34–13.37)	**0.007**
IgM (g/l)	1.04 (0.74–1.40)	1.17 (0.77–1.66)	0.273
Microbiological positivity (overall)–no. (%)	95 (36.8)	28 (65.1)	** < 0.001**
*S.aureus*	59 (22.9)	12 (27.9)	0.471
MRSA	5 (1.9)	4 (9.3)	**0.027**
CNS^a^	6 (2.3)	3 (6.9)	0.123
*K.kingae*	6 (2.3)	0 (0)	0.599
Mycobacteria spp.	2 (0.8)	4 (9.3)	**0.004**
Local complications			
Perilesional cellulitis–no. (%)	88 (34.1)	20 (46.5)	0.116
Myositis–no. (%)	51 (19.8)	10 (23.3)	0.598
Soft-tissue abscess–no. (%)	105 (40.7)	30 (69.8)	** < 0.001**
Bone sequestration–no. (%)	29 (11.2)	9 (20.9)	0.077
**Antibiotic therapy–median (Q1–Q3)**			
IV therapy (days)	21 (14–28)	28 (14–46)	**0.015**
OS therapy (days)	16 (12–24)	17 (14–35)	0.345
Total antibiotic therapy (days)	40 (28–49)	51 (39–81)	** < 0.001**
**Surgical intervention**			
Curettage–no. (%)	23 (8.9)	16 (37.2)	** < 0.001**
Arthrocentesis–no. (%)	6 (2.3)	4 (9.3)	**0.040**
Osteosynthesis–no. (%)	11 (4.3)	1 (2.3)	1.000
Hospitalization (days)–median (Q1–Q3)	22 (15–30)	31 (17–54)	**0.005**
Follow-up (months)–median (Q1–Q3)	8 (4–14)	9 (6–15)	0.200
Relapse–no. (%)	13 (5.0)	4 (9.3)	0.280
Chronic osteomyelitis–no. (%)	29 (11.2)	16 (37.2)	** < 0.001**

*Percentages are calculated accounting for missing values.

Significant values are in bold

The variables that differed significantly in the two groups were, respectively: presence of sepsis (34.9% vs 14.0%, *p* = 0.001); median IgG levels (10.6 vs 8.94 g/l, *p* = 0. 007); microbiological positivity (65.1% vs 36.8%, *p* < 0.001); MRSA infection (27.9% vs 22.9%, *p* = 0.027); mycobacterial spp. infection (9.3% vs 0.8%, *p* = 0.004); presence of a soft tissue abscess (69.8% vs 40.7%, *p* = 0.004). 8% vs 40.7%, *p* < 0.001); median duration of IV (28 vs 21 days, *p* = 0.015) and median duration of total antibiotic therapy (51 vs 40 days, *p* < 0.001); need for surgery with curettage (37. 2% vs 8.9%, *p* < 0.004). 2% vs 8.9%, *p* < 0.001) or arthrocentesis (9.3% vs 2.3%, *p* = 0.040); median length of hospitalization (31 vs 22 days, *p* = 0.005); presence of chronic osteomyelitis (37.2% vs 11.2%, *p* < 0.001). Adjusting all significant variables for each other in the logistic regression analysis, as shown in Table 4, only IgG levels (OR 1.11, *p* = 0.034) and duration of intravenous antibiotic therapy (OR 1.038, *p* = 0.006) remained valid predictors of sequelae. In the final model, the presence of sepsis at onset also contributed to the development of sequelae (OR 2.428), although not significantly (*p* = 0.059). ROC curve analysis did not yield a valid cutoff of IgG levels to discriminate the presence or absence of sequelae (AUC 65.3%, data not shown).

**Table 4:** Logistic regression (dependent variable: sequelae).

	Odds ratio	C.I. (95%)	*p-value*
Sepsis (yes)	2.428	0.967 – 6.098	0.059
IgG (g/l)	1.110	1.008 – 1.223	**0.034**
IV therapy (days)	1.038	1.011 – 1.065	**0.006**

Significant values are in bold

## Discussion

In our article, we reported relevant clinical data from one of the largest cohorts of children with osteomyelitis described in the literature. We observed an even distribution of patients by age and sex, with a slight prevalence of older age and males, demonstrating that acute hematogenous osteomyelitis is a disease that can affect the entire pediatric population. Most patients were hospitalized within 2 weeks after the onset of fever, swelling and heat at the affected site and treated with antibiotic therapy as soon as cultures were sent for microbiological analysis. In our sample, fever at the time of admission was present in only 45% of the children, which was lower than the 61.7% reported by Dartnell et al.^8^.

Similar to previous studies^17,18^, in our sample we found an elevated ESR, while CRP and white blood cell count were slightly or not at all elevated at the time of diagnosis, confirming a low sensitivity of the latter tests in the diagnosis and follow-up of the disease. Most patients underwent radiography and/or MRI of the affected site to clearly diagnose and define the extent and severity of bone infection. Although not recommended for the diagnosis of pediatric bacterial osteomyelitis^9^, we suggest the use of ultrasound to rule out suspected joint and skin spread of primary bone infection whenever clinical evidence of disease progression to adjacent tissues is present. In our sample, 57.1% of patients underwent joint ultrasound, 47.3% of whom were diagnosed with concomitant arthritis. In addition, ultrasonography can identify soft tissue abscesses (43.9% of our sample) adjacent to the bone focus. Ultrasound-guided aspiration of these foci can also guide the diagnosis and management of pediatric bacterial osteomyelitis shortly after admission^9^, before more advanced imaging is available. In our sample, more than one-third of cultures (40.1%) were positive for any pathogen, consistent with positivity rates reported in the literature^8^. The most commonly isolated pathogen was methicillin-sensitive *S.aureus*, followed by MRSA and coagulase-negative staphylococci. Therefore, the most commonly prescribed antibiotics were broad-spectrum cephalosporins and linezolid, the latter a gram-positive antibiotic that reaches high concentrations within the infected bone^19^, followed by glycopeptides. Like linezolid, quinolones also have good bone penetration and were therefore generously prescribed in the outpatient setting when age was not a questionable limitation to their administration^20^. Given the frequent staphylococcal etiology in culture-positive cases, culture-negative osteomyelitis has been treated as a presumed gram-positive disease^21^, with good results in long-term follow-up. The use of second-line antibiotics was justified by the high prevalence of MRSA infections in our hospital (> 10% of isolates), the need to treat complicated osteomyelitis when previous antibiotic regimens had failed, and the lack of supply of more common antibiotics to our pharmacy due to the more frequent use of aggressive antibiotic therapy in a tertiary care center. Given the frequent isolation of MRSA, we suggest aggressive empiric antibiotic treatment with bactericidal agents (such as glycopeptides, aminoglycosides, and linezolid) if the local prevalence of MRSA infection is high. With a careful antibiotic stewardship program, most patients avoided surgery and were hospitalized for less than one month, with a late sequelae rate of 13.5%. Comparing patients with or without sequelae, we found a significant prevalence of factors consistent with increased virulence and spread of the infectious agent within the bone and beyond, such as: sepsis and microbiological positivity (with MRSA and mycobacterial etiology more common); higher IgG levels; and signs of chronic osteomyelitis. Not surprisingly, a higher prevalence of sequelae was observed in patients with longer antibiotic therapy, length of stay, and need for surgery or arthrocentesis. However, adjusting all factors for each other in multivariate analysis, only IgG levels (*p* = 0.034) and duration of intravenous antibiotic therapy (*p* = 0.006) remained good predictors of sequelae. The presence of sepsis at onset was just above the statistically significant threshold (*p* = 0.059). Of note, prolonged antibiotic therapy no longer correlated with the development of sequelae in multivariate analysis, consistent with recent guidelines on the management of uncomplicated acute hematogenous hematogenous osteomyelitis in children, which suggest reduced antibiotic therapy with early switch to oral antibiotics guided by clinical and laboratory data^13^.

Our results indicate that assaying IgG levels may be useful in identifying children with pediatric bacterial osteomyelitis at increased risk of developing sequelae, as well as those with latent immunodeficiencies that may have caused or aggravated the disease. Although immunoglobulin levels were not correlated with age in our sample (Fig. 1), we recommend adopting age-adjusted reference intervals for their assessment^22^.

Our study is limited by its retrospective nature. Therefore, further studies are needed to confirm or refute our findings before we can generalize and apply them to the entire population of children with pediatric bacterial osteomyelitis. In addition, because our study covers a long period of time, we may have underestimated the prevalence of some infections that are easily diagnosed today, as in the case of K.kingae infection, for which species-specific real-time PCRs are recently available. In addition, we failed to follow many patients up to the recommended limit of 12 months post-infection, mainly because of the refusal to comply with the visit schedule when patients were feeling well.

## Conclusion

In this study we report a sample of children with pediatric bacterial osteomyelitis who were hospitalized over a period of 11 years. Our results show that a severe presentation with sepsis and hypergammaglobulinemia at onset is associated with a higher incidence of sequelae. In this context, assaying IgG levels may be a valuable adjunct to the laboratory workup of these children to guide the clinician toward prompt recognition and treatment of pediatric bacterial osteomyelitis.

## Data Availability

The datasets used and/or analyzed during the current study available from the corresponding author on reasonable request.

## Abbreviations

CBC: Complete blood count
CRP: C-reactive protein
ERS: Erythrocyte sedimentation rate
IV: Intravenous administration
MRI: Magnetic resonance imaging
MRSA: Methicillin-resistant staphylococcus aureus
OS: Oral administration
